# Miscellaneous Items

**Published:** 1886-01

**Authors:** 


### MISCELLANEOUS ITEMS

**Did** the patients of Job ever get better?

**When** Eve presented Adam with a little Cain did he use it as a walking stick, or did he wait until he got Abel?

**Our Long Articles** crowded us more than we intended, but they are all important and will be read with interest.

**Six** doctors were attending the king of Spain when he died. Is it any wonder that the poor fellow died?

**S. M.**—Articles on anything that improves the health or prevents illness are always acceptable.

**G. B.**—Don't be afraid of drinking mox ye. It will not hurt you. We could take a pint, as well as half that quantity, if we could only hold it. Its perfectly innocent except to the pocket.

**F. R. S.**—We have none of the old volumes of the JOURNAL OF HEALTH. That was a part of the assets we did not purchase. We begin with Jan. 1st.

**President Hopkins** says he has little respect for the emotional religious people, who groan and shout in their meetings, and steal chickens on their way home.

**Medical** science, if, indeed, there is any such science, is little more than a classification of remedial discoveries made by empirics. —*Chicago Times.*

**J. S. Grant,** of Salem, Mo., says he has tried Tongaline for asthma, which has proved perfectly successful. He commends it as a good medicine for that troublesome complaint.

**Dr. W. R. Chittich** urges the use of oxalate cerium in much larger doses than those usually given for the arrest of vomiting. He would give eight or ten grains every two hours.

**In** Spain, up to the close of the week ending Oct. 21st, 273,549 cases of cholera had been reported, with a mortality of 101,346. In Italy, there were 1,375 cases, and 689 deaths.

**Powdered Resin** is the best thing to stop bleeding from cuts. After the powder is sprinkled on, wrap the wound with a soft cotton cloth. As soon as the wound begins to feel feverish, keep the cloth wet with cold water.

**Make Remittances.**—In postal notes or express notes if possible, for small amounts. No postal stamps are current with us for more than two cents. Do not send in any of the 5 or 10 cent. We have more than we can use of these denominations and cannot afford to sell them at a discount after deducting for agents.

**Scarlet** fever of a malignant type, has broken out at the Protestant Episcopal Home for children at Pittsburg. There are eighty-five children in the home and the situation is alarming. On the 16th four deaths had occurred and ten children were lying ill with the dread disease.

**A solution** of methol is proving a useful and comparatively an inexpensive substitute for cocoaine in cases requiring local anaesthesia of the mucous membrane of the nose, pharynx, larynx, etc. It is reported to be more transitory in its effect than cocoaine, and to possess cumulative action, but that remains to be proved yet.

**We** learn by a telegram from Evansville, Ind., that a very remarkable operation has been performed at the City Hospital. Joseph Whye has had his left kidney removed, it having been rendered useless by an abscess, and filled with bloody puss. He endured the operation well, and seemed to have a chance for life.

**Danger From Fig Tree Twigs.**—The Santa Cruz *Surf* says: A few weeks ago a little child of Seth Blanchard's was playing with some broken twigs from a fig tree and got some of the sap which exuded therefrom in her eyes. Her eyes became badly inflamed and it is reported that for some time the child has been entirely unable to see.

**Typhoid Fever** has had and is having quite a run in Brooklyn, and it appears in some of the best houses and families in the city; but this does not affect our very clear apprehension of the cause. It is not providential but is a decided improvidence somewhere, and there should be no hesitation in searching for the causes. We trust it has had its worst run.

**The Hog.**—Seventeen persons in this city were seized with trichinosis, the result, it is stated, of eating ham that was not cooked enough to kill the living animals. If you eat pork you cannot be too careful about the cooking; have it thoroughly cooked, so that the animals will surely be all done to death. It is the only safe thing to do with pork unless it be to do nothing.

**The New London** (Conn.) *Telegraph* notices a singular accident at the Catholic church of St. Mary, Star of the Sea, in that city. Patrick Cooney, one of the collectors had taken up the collection in the main aisles, made the usual genuflections before the altar previous to passing to the side aisles. In kneeling, the muscle attached to the knee-pan on both legs were ruptured, and before he reached the side aisles he fell and was unable to rise. The physicians are of the opinion that he will be confined to his house for at least three months, and fear that he may never recover the full use of his legs.

**A Learned Physician.**—A paper at Lancaster, Pa., recently published an article from an old and leading physician, one who has practiced there for half a century, and has a great reputation in that State, giving an account of cocaine and its properties. He describes the *theobroma cocoa* and stated the good effect of the drinks, as choclate cocoa, etc., and said that the wonderful effects had been discovered in the *bean* of this tree, as a medicine. So he put himself on record. What was his surprise to find that he was wholly mistaken by the name that is something like cocoa, but that is not cocaine by a great deal the latter being a bitter shrub.

**Tennessee.**—The state Board of Health publishes a Monthly Bulletin, which is a good example for every State. It urges the people to be active in vaccination for the prevention of small-pox while there is no case in the state, but such preventions are always in order. The bulletin says a large portion of the people are not vaccinated. We are most happy to be able to state that:

"One question, however, in which the Tennessee State Board is justly taking an active interest, that of prison construction and prison management, was the subject of intelligent papers and profitable discussion by the most learned experts and philanthropists of the country. Also the question of the care and management of the insane."

**For Frost-Bitten Toes.**—Dr. Lapatin, in the "Proceedings of the Caucasian Medical Society," advises that the fingers and toes which have been slightly frost-bitten, and which consequently suffer from burning, itching and pricking sensations, should be painted, at first once, and afterwards twice a day, with a mixture of dilute nitric acid and peppermint water in equal proportions. After this application has been made for three or four days, the skin becomes darkened with the epidermis is shed, healthy skin appearing under it. The cure is effected in from ten to fourteen days. The author has found this plan very effectual amongst soldiers, who were unable to were their boots in consequence of having had frozen feet. They were in this way, soon rendered capable of returning to duty.

